# Characterization of immunoglobulin loci in *Pongo abelii* and *Pongo pygmaeus*: Insights from multi-genome annotation and IMGT-based curation

**DOI:** 10.1093/nargab/lqag112

**Published:** 2026-09-28

**Authors:** Shamsa Batool, Guilhem Zeitoun, Chahrazed Debbagh, Boutayna Dhioui, Géraldine Folch, Joumana Jabado-Michaloud, Véronique Giudicelli, Sofia Kossida

**Affiliations:** IMGT®, The International ImMunoGeneTics Information System®, Institute of Human Genetics (IGH), National Center for ScientificResearch (CNRS), University of Montpellier (UM), 34000 Montpellier, France; IMGT®, The International ImMunoGeneTics Information System®, Institute of Human Genetics (IGH), National Center for ScientificResearch (CNRS), University of Montpellier (UM), 34000 Montpellier, France; IMGT®, The International ImMunoGeneTics Information System®, Institute of Human Genetics (IGH), National Center for ScientificResearch (CNRS), University of Montpellier (UM), 34000 Montpellier, France; IMGT®, The International ImMunoGeneTics Information System®, Institute of Human Genetics (IGH), National Center for ScientificResearch (CNRS), University of Montpellier (UM), 34000 Montpellier, France; IMGT®, The International ImMunoGeneTics Information System®, Institute of Human Genetics (IGH), National Center for ScientificResearch (CNRS), University of Montpellier (UM), 34000 Montpellier, France; IMGT®, The International ImMunoGeneTics Information System®, Institute of Human Genetics (IGH), National Center for ScientificResearch (CNRS), University of Montpellier (UM), 34000 Montpellier, France; IMGT®, The International ImMunoGeneTics Information System®, Institute of Human Genetics (IGH), National Center for ScientificResearch (CNRS), University of Montpellier (UM), 34000 Montpellier, France; IMGT®, The International ImMunoGeneTics Information System®, Institute of Human Genetics (IGH), National Center for ScientificResearch (CNRS), University of Montpellier (UM), 34000 Montpellier, France; Institut Universitaire de France (IUF), 75231 Paris Cedex 05, France

## Abstract

Antibodies, or immunoglobulins (IGs), are central to the vertebrate adaptive immune system, yet the genomic architecture of IG loci remains poorly characterized in many nonhuman primates. In this study, we present the first comprehensive genomic analysis of the IG loci (IGH, IGL, and IGK) in two critically endangered orangutan species; *Pongo abelii* (Sumatran orangutan) and *Pongo pygmaeus* (Bornean orangutan) across seven genome assemblies. Using IMGT-standardized biocuration framework combined with read-level structural validation, we identified previously undocumented haplotype-specific variation, including multigene duplications and deletions, and expansions of variable gene families within the analyzed assemblies. Recombination signal sequence and switch region analyses revealed conserved regulatory motifs with potential implications for V(D)J recombination and class-switch recombination. These findings underscore the complexity and evolutionary adaptability of IG loci in great apes and highlight the value of orangutans as key references for understanding immune system evolution in the Hominidae lineage.

## Introduction

Antibodies or immunoglobulins (IGs) are the main molecules expressed by B cells mediating the adaptive humoral immune response. IGs are antigen receptors present in all gnathostomes (jawed vertebrates); they recognize and neutralize pathogens through a variety of mechanisms, including blocking infection and facilitating pathogen clearance [1]. They comprise four polypeptide chains: two identical heavy (H) chains and two identical light IG chains, which may be lambda (λ) or kappa (κ). The N-terminal region, or Fab (fragment antigen-binding), is extremely diverse and responsible for recognizing and interacting with the antigen, while the C-terminal region, or Fc (fragment crystallizable, the “antibody tail”), is responsible for triggering the effector B cell responses. In placental mammals (eutheria), five classes of IGs are known to exist: IGHM, IGHD, IGHG, IGHE, and IGHA, each encoding a different class of IG [2, 3]. They perform various effector functions, such as complement binding, binding to phagocytic cells, opsonization, and transport across the placental epithelium [1]. Antibodies are not directly encoded in the germline genome; instead, they are produced through somatic V(D)J recombination processes, a site-specific recombination process that joins variable (V), diversity (D), and joining (J) genes to generate antibody diversity [4]. V(D)J rearrangements are facilitated by the recombination activating gene (RAG) proteins, RAG-1 and RAG-2. These proteins are responsible for recognizing and cleaving DNA at specific sites, producing precise double-strand breaks at the junctions between the recombination signal sequences (RSSs) and the coding regions [5]. This process affects the IG loci that contain variable (V), diversity (D), and joining (J) genes. In the heavy chain, recombination occurs in two steps: first, the D and J genes are rearranged, followed by the second rearrangement with a V gene to form the complete V(D)J exon that encodes the variable domain of the antibody. In contrast, light chains (kappa or lambda) lack D genes and are assembled by recombining only V and J genes. After antigen recognition, in order to enhance the affinity, the antibodies are then diversified by somatic hypermutation, which introduces point mutations in certain hotspots [6]. IG loci have diversified rapidly and independently across jawed vertebrate lineages, resulting in major differences in their genomic organization, gene content, and the proportion of functional genes versus pseudogenes, reflecting strong lineage-specific evolutionary pressures [2].

IMGT®, the international ImMunoGeneTics information system® [7] established in 1989, remains the global reference framework for immunogenetics and immunoinformatics. By providing standardized ontology, nomenclature, and annotation rules for IG and T cell receptor (TR) genes, IMGT enables consistent characterization of adaptive immune loci across species [9, 10]. Since its creation, continuous biocuration efforts have progressively expanded IMGT® reference directories through the annotation of numerous vertebrate species [11–14], while similar work remains ongoing for many others. Together, these efforts support comparative immunology, evolutionary analyses, and studies of immune gene diversity and function.

Nonhuman primates are of great interest in comparative studies and biomedical research due to their close similarities with humans, providing important insights into the evolutionary processes that have shaped genome structure, function, and genetic diversity [15]. Several studies have explored the relationship between nonhuman primate evolution and human disease, with a particular focus on segmental duplications observed in great apes and humans, which are thought to play an important role in human disease susceptibility, as in the case of their impact on genes associated with Mendelian diseases [16, 17]. Studies of adaptive immune responses across various vertebrate species open new therapeutic opportunities [18]. Gaining insight into the diversity of adaptive immune systems across different vertebrates can also aid in examining the transmission and spread of novel zoonotic pathogens [19, 20].

Orangutans were once classified into two subspecies. However, recent research and taxonomic revisions, driven by extensive molecular and morphological analyses, have shown that the genetic and physical differences between Sumatran and Bornean orangutans are as significant as, or even greater than, those between the two chimpanzee species, the common chimpanzee and the bonobo [21–27]. As a result, updated classifications now recognize Sumatran and Bornean orangutans as distinct species; *Pongo abelii* in Sumatra and *Pongo pygmaeus* in Borneo, with an estimated divergence time of around 1.1 million years ago [25, 28]. However, dissenting opinions exist [29], noting that despite a pericentric inversion in chromosome 2, interbreeding between these groups in captivity can still produce fertile offspring [30]. A third species, *Pongo tapanuliensis*, identified in the Batang Toru region of Sumatra, diverged from *Pongo abelii* around 3.4 million years ago based on genomic and morphological data [31]. Because orangutans (genus *Pongo*), the only Asian great apes, are the most distantly related to humans within the Hominidae family, they hold particular significance in studies exploring evolutionary relationships among great apes. The orangutan genome was published in 2011 [32], revealing a strikingly slower rate of structural genome evolution in the *Pongo* lineage. Assuming consistent mutation rates and generation times across the Hominidae family, the most recent common ancestor of orangutans and the other great apes is estimated to have lived between 15 and 21 mya [28, 33–35].

Our analysis of IG loci in two orangutan species (*Pongo abelii* and *Pongo pygmaeus*), aiming to identify both shared and distinct features between these two species and humans provides valuable insights into the evolution of immune response mechanisms and immune system adaptability across great apes. While great apes represent the most appropriate model for evaluating the safety and efficacy of vaccines and treatments intended for human use, concerns regarding endangered species status, cost, and limited availability pose significant ethical and practical challenges and make the use of great apes, including orangutans, highly restricted [36]. Nevertheless, genomic data from these species remain essential in experimental research for advancing our understanding of immunological diseases and supporting the development of potential therapies. This information can provide insights into the differences in primate immune systems and how they have evolved to respond to infections. Likewise, it should have implications for biomedical trials for drug development and evaluation [37].

## Materials and methods

### Genomic dataset selection and preliminary assessment

To investigate the IG loci in orangutan genomes, we analyzed all genome assemblies (*n* = 13) available in the NCBI Genome database. These assemblies represented two orangutan species (*Pongo abelii* and *Pongo pygmaeus*), and seven assemblies were selected using IMGT’s predefined two-phase pre-selection criteria Supplementary Table S1 [14].

### Long read based assembly validation

Following selection, we used IMGT/StatAssembly (version v1.0.2) [7, 8] as part of the IMGT-developed quality control process, incorporating long-read sequencing data retrieved from NCBI BioProjects. Preference was given to HiFi reads due to their medium read length but high accuracy; when HiFi data were unavailable, PacBio CCS or the highest-quality long reads were used. Raw reads underwent quality assessment using FastQC [38], followed by locus-specific alignment using minimap2 (2.27-r1193) [39] with the -x map-hifi preset. The BAM file was generated from reads based on the script (https://src.koda.cnrs.fr/imgt-igh/statassembly). This workflow ensured high-confidence, structurally validated assemblies for downstream IG gene analysis.

### IMGT locus extraction, gene annotation, functionality determination, and database integration

Locus extraction, gene identification, and annotation were performed according to the IMGT biocuration framework [13, 14, 40]. Gene characterization included the identification of V (variable), D (diversity), J (joining), and C (constant) genes, determination of gene functionality, validation of coding regions, and conserved immunogenetic features. Gene and allele names were assigned according to the IMGT standardized nomenclature rules (accessible via https://www.imgt.org/IMGTScientificChart/Nomenclature/IMGTnomenclature.php). Annotated genes and alleles were subsequently integrated into the corresponding IMGT databases and reference directories. The annotated locus sequences were also submitted to the NCBI Third Party Annotation (TPA) [41], and TPA accession numbers were assigned (https://www.imgt.org/IMGTrepertoire/numacc.php). This dual referencing ensures full correspondence between IMGT and NCBI records, facilitating transparent cross-referencing and traceability of annotated IG loci across databases.

### Validation of novel alleles and structural variants

Given that the IG loci are highly complex and polymorphic genomic regions enriched with segmental duplications and highly similar gene families, they are prone to misassemblies or collapsed regions in standard genome assemblies [42]. To resolve these complexities and to validate haplotype-specific duplications and insertions identified in the IG loci, we performed read-level analysis. Using IMGT/StatAssembly, BAM alignments of HiFi retrieved from the NCBI BioProjects PRJNA916742 and PRJNA916743 were used to assess read coverage and consistency across exonic regions. Regions containing genes present exclusively on one haplotype were validated by aligning raw Nanopore reads back to the corresponding assemblies and visualizing these regions in IGV (Integrative Genomics Viewer v2.18.5 + dfsg-1). For each candidate duplication or insertion, we confirmed visually the presence of continuous long reads spanning the variant region, providing strong evidence for its authenticity. This approach ensured that both novel alleles and observed haplotype-specific gene gains were supported by the underlying read data and were not artifacts of genome assembly or misalignment.

Alleles and genes that failed to meet validation standards were excluded or retracted, with data updates tracked in IMGT/GENE-DB (available at https://www.imgt.org/IMGTgenedbdoc/dataupdates.html).

### Analysis of RSS

For functional IG V, D, and J genes, the corresponding RSSs were extracted from the IMGT database for the IGH, IGK, and IGL loci to assess the degree of conservation and variation of RSSs in *Pongo* species [13]. These sequences include the conserved heptamer and nonamer motifs separated by a spacer typically 12 or 23 base pairs (bp), flanking the coding segments, which are essential for RAG-mediated V(D)J recombination [5]. Sequence logos were generated using WebLogo3 (v3.9.0) using probability matrices [43]. Spacer length distributions were shown between the motif panels. For each RSS element (V-HEPTAMER, V-NONAMER, J-HEPTAMER, and J-NONAMER), the locus-specific consensus sequence was determined. The percentage identity of each RSS sequence relative to its corresponding locus-specific consensus sequence was calculated to compare the degree of RSS conservation among the IGH, IGK, and IGL loci. In addition, the orangutan RSS consensus sequences were compared with the corresponding human functional RSS consensus sequences.

The percentage identity of each RSS sequence relative to its corresponding locus-specific consensus sequence was calculated, and the distributions among the IGH, IGK, and IGL loci were compared using the Kruskal–Wallis test followed by Dunn’s post-hoc pairwise comparisons with Benjamini–Hochberg correction for multiple testing. Statistical significance was defined as an adjusted *P* value < .05.

### Identification and analysis of switch regions

To investigate the organization and conservation of potential IG switch (S) regions in orangutans, genomic regions upstream of each constant region (IGHC) gene were examined for characteristic sequence features associated with class-switch recombination (CSR). Coordinates of IGHC genes were obtained from annotated genome assemblies of *Pongo abelii* and *Pongo pygmaeus* (GCF_028885655.2 & GCF_028885625.2 respectively). For each IGHC gene, a 5-kb genomic sequence upstream of the 5′ end of the first CH1 exon, as defined by the IMGT-curated gene annotation, was extracted from the indexed genome using BEDTools *getfasta*, taking strand orientation into account. The 5′ end of the CH1 exon was used as the reference point for all IGHC genes to ensure a consistent definition of the upstream region for comparative analysis. These upstream regions, expected to encompass the IG switch (S) regions, were screened for degenerate pentamer motifs (GAGCW, where W = A or T; and GGGBT, where B = C, G, or T) representing canonical AID-target sequences [44, 45]. Motif counts and GC content were computed using custom Python scripts and normalized per kilobase to allow comparison among genes. Local repeat structures within each upstream region and across the entire IGHC cluster were visualized by self-dotplot analysis in standalone Gepard application [46] using a word size of 14.

### Comparative and phylogenetic analysis of the immunoglobulin loci across four species

IG variable (V) gene clusters (V-CLUSTER) for the heavy (IGHV) and light chains (IGKV, IGLV) were analyzed across multiple genome assemblies of *Homo sapiens, Pongo abelii, Pongo pygmaeus*, and *Gorilla gorilla gorilla*. For each genome, the length of each V-CLUSTER (in base pairs) and the number of V genes were recorded. Gene density was calculated as the number of V genes per megabase (genes/Mb) of the cluster using the formula:


\begin{eqnarray*}
\mathrm{Gene}\,\,\mathrm{density}\left( {\mathrm{genes}/\mathrm{ Mb}} \right) = \frac{{\left( {\mathrm{Number}\,\,\text{of V}\,\,\mathrm{genes} \times {{10}^6}} \right)}}{{V - \mathrm{Cluster}\,\,\mathrm{length}\left(\text {bp} \right)}}.
\end{eqnarray*}

Densities were calculated separately for each genome assembly and then averaged across assemblies within each species to obtain mean values and standard deviations for each locus. Comparative analyses and visualizations were performed in Python using *pandas* and *matplotlib*. Locus size versus gene number relationships were visualized as scatterplots, and species-level summaries as bar charts showing mean density ± SD.

Phylogenetic relationships among IG variable (V) genes from the IGHV, IGKV, and IGLV loci of *Homo sapiens, Pongo abelii, Pongo pygmaeus*, and *Gorilla gorilla gorilla* were analyzed using NGPhylogeny “One Click” online workflow [47]. All annotated V genes were represented by their 01 alleles (IMGT reference sequences). The pipeline performs multiple sequence alignment with MUSCLE [48] and constructs maximum-likelihood trees using PhyML [49]. Bootstrap support values (100 replicates) were calculated automatically, and resulting trees were visualized in iTOL [50] using circular layouts. Branches were color-coded by species, and color strips indicated IGHV, IGKV, and IGLV subgroup classifications.

## Results

### Dataset selection

Table 1 provides a comprehensive summary of the information of the selected assemblies, including locus positions and corresponding accession numbers within the IMGT/LIGM-DB (v1.2.11).

**Table 1.** Summary of genome assemblies and IG loci annotated in *Pongo* species

Genome assembly	Isolate	GenBank assembly ID	Chromosome GenBank assembly ID/scaffold ID	Chromosome GenBank assembly/ Contig locus positions	Locus length (bp)	IMGT locus orientation on chromosome	IMGT/LIGM-DB /TPA accession numbers
IGH on chromosome 14/15^a^							
*Pongo abelii* (Sumatran orangutan), NCBI taxon:9601							
Susie_PABv2^b^	Susie	GCA_002880775.3	CM009276.2	87402090..88963417	1561328	REVERSE	IMGT000122 BK075454
NHGRI_mPonAbe1-v2.1_pri	AG06213	GCA_028885655.3	NC_072000.2	110080644..103733284	1649699	REVERSE	IMGT000206 BK075456
NHGRI_mPonAbe1-v2.0_alt	AG06213	GCA_028885685.2	CM054671.2	102190285..103682647	1543000	REVERSE	IMGT000121 BK075455
Susie_PAB_hifiasm-v0.15.2.pri	Susie	GCA_030170355.1	JARRAK010000025.1	89157563..90724507	1566945	–	IMGT000157
Susie_PAB_hifiasm-v0.15.2.alt	Susie	GCA_030170345.1	JARRAL010000083.1	27984844..27999048	1518880	–	IMGT000167
*Pongo pygmaeus* (Bornean orangutan), NCBI taxon:9600							
NHGRI_mPonPyg2-v2.1_pri	AG05252	GCA_028885625.3	NC_072388.2	104726350..106399170	1745983	REVERSE	IMGT000230 BK075458
NHGRI_mPonPyg2-v2.0_alt	AG05252	GCA_028885525.2	CM054622.2	105311893..107057879	1672821	REVERSE	IMGT000229 BK075457
IGL locus on chromosome 22/23^a^							
*Pongo abelii* (Sumatran orangutan), NCBI taxon:9601							
Susie_PABv2^b^	Susie	GCA_002880775.3	CM009284.2	3326936–4190098	863163	REVERSE	IMGT000108 BK075449
NHGRI_mPonAbe1-v2.1_pri	AG06213	GCA_028885655.3	NC_085929.1	25547691..26403226	855536	REVERSE	IMGT000246 BK075450
NHGRI_mPonAbe1-v2.0_alt	AG06213	GCA_028885685.2	CM068846.1	21999085..22854931	855847	REVERSE	IMGT000247 BK075451
Susie_PAB_hifiasm-v0.15.2.pri	Susie	GCA_030170355.1	JARRAK010000033.1	17758621..18622348	150504	–	IMGT000189
Susie_PAB_hifiasm-v0.15.2.alt	Susie	GCA_030170345.1	JARRAL010000044.1	31155160..32018390	950543	–	IMGT000190
*Pongo pygmaeus* (Bornean orangutan), NCBI taxon:9600							
NHGRI_mPonPyg2-v2.1_pri	AG05252	GCA_028885625.3	NC_085931.1	29525211..30907261	1382051	REVERSE	IMGT000278 BK075453
NHGRI_mPonPyg2-v2.0_alt	AG05252	GCA_028885525.2	CM068910.1	20975388..22354260	1378873	REVERSE	IMGT000248 BK075452
IGK locus on chromosome 2A/12^a^							
*Pongo abelii* (Sumatran orangutan), NCBI taxon:9601							
Susie_PABv2^b^	Susie	GCA_002880775.3	CM009263.2	19648734–20476838	828105	FORWARD	IMGT000096 BK063599
NHGRI_mPonAbe1-v2.1_pri	AG06213	GCA_028885655.3	NC_071997.2	43451819–44286661	834843	FORWARD	IMGT000244 BK075446
NHGRI_mPonAbe1-v2.0_alt	AG06213	GCA_028885685.2	CM054668.2	42039675..42823442	783768	FORWARD	IMGT000245 BK075447
Susie_PAB_hifiasm-v0.15.2.pri	Susie	GCA_030170355.1	JARRAK010000016.1	80163254..80977396	814143	–	IMGT000201
Susie_PAB_hifiasm-v0.15.2.alt	Susie	GCA_030170345.1	JARRAL010000033.1	15005270..15802831	797562	–	IMGT000202
*Pongo pygmaeus* (Bornean orangutan), NCBI taxon:9600							
NHGRI_mPonPyg2-v2.1_pri	AG05252	GCA_028885625.3	NC_072385.2	44675560..45472918	797359	FORWARD	IMGT000275 BK075722
NHGRI_mPonPyg2-v2.0_alt	AG05252	GCA_028885525.2	CM054619.2	47958023..48790500	832478	FORWARD	IMGT000276 BK075448

a Chromosome numbers differ in the latest telomere-to-telomere (T2T) assemblies due to updated chromosome nomenclature and reannotation of genomic structure (https://ncbiinsights.ncbi.nlm.nih.gov/2024/05/13/refseq-release-224/).

b Reference assembly for *Pongo abelii* in IMGT databases.

This table summarizes the annotated genome assemblies of *Pongo abelii* (Sumatran orangutan) and *Pongo pygmaeus* (Bornean orangutan) and the corresponding immunoglobulin heavy (IGH), lambda light (IGL), and kappa light (IGK) chain loci identified within them. For each assembly, the GenBank accession identifiers, chromosomal or scaffold coordinates, and locus orientations are provided along with the associated IMGT/LIGM-DB accession numbers.

All genome assemblies used for the annotation of IG loci were subjected to the IMGT quality control process (Supplementary Table S1). Assemblies meeting the established quality criteria were retained for downstream annotation and comparative analyses of the IGH, IGL, and IGK loci (Supplementary Figs S1 and S2).

#### Susie

For the *Pongo abelii* individual Susie, we analyzed three available genome assemblies: Susie_PABv2 (GCA_002880775.3), Susie_PAB_hifiasm-v0.15.2.pri (GCA_030170355.1), and Susie_PAB_hifiasm-v0.15.2.alt (GCA_030170345.1). The Susie_PABv2, released in 2018, serves as the reference assembly in the IMGT database and represents a more complete, chromosome-scale sequence, while the latter two released in 2023 generated using the Hifiasm assembler, correspond to the primary and alternate haplotypes, respectively, and are estimated to be more accurate (quality value [QV] = 42–58 or 99.9937%–99.9998% accuracy) and significantly more contiguous (contig N50 = 19–104 Mbp) [51]. The Hifiasm-based assemblies do not fully meet IMGT quality criteria due to incomplete contiguity and lack of full scaffolding and assembly polishing (Supplementary Table S1). Nevertheless, they offer valuable phased views of the IG loci, enabling detection of allelic variation, hemizygosity, and haplotype-specific structural events that are not readily captured in the haploid representation of Susie_PABv2. By analyzing and annotating IG genes across all three assemblies, we utilized the high continuity of the reference genome and the resolution of haplotype-specific variation achieved through long-read phasing to obtain a more complete understanding of IG diversity in this individual.

#### Telomere to telomere assemblies alter chromosomal localization of IG loci

We analyzed four telomere-to-telomere (T2T) assemblies released in 2024, NHGRI_mPonPyg2-v2.1_pri (GCF_028885625.2), NHGRI_mPonPyg2-v2.1_alt (GCA_028885525.2), NHGRI_mPonAbe1-v2.1_pri (GCF_028885655.2), and NHGRI_mPonAbe1-v2.1_alt (GCA_028885685.2) representing phased genome sequences for *Pongo pygmaeus* (AG05252) and *Pongo abelii* (AG06213). These assemblies employ a revised chromosome numbering convention based on alignment to a common great ape and human reference, resulting in reassigned chromosome identifiers better reflecting evolutionary and structural correspondence [52]. For instance, the IGH locus, previously annotated on chromosome 14, is now located on chromosome 15; the IGK locus shifts from chromosome 2A to 12; and the IGL locus from 22 to 23. This renumbering stems from updated assembly orientation and synteny mapping, aiming to harmonize chromosome identities across species for comparative genomics (e.g. aligning acrocentric chromosomes consistently across primates) through chromosome assignments based on inferred homology relationships and correspondence to the ancestral great ape karyotype. Accordingly, chromosome identifiers differ from those reported in earlier assemblies and publications, and correspondence between the two numbering systems is required when comparing genomic locations [52, 53].

Despite the availability of newer T2T genome assemblies for *Pongo abelii* and *Pongo pygmaeus*, we have retained Susie_PABv2 and its corresponding chromosome numbering (14 for IGH, 2A for IGK, and 22 for IGL) as the reference framework for IG loci annotation in the IMGT database. Susie_PABv2 remains a widely used reference for *Pongo abelii* [54–56], and it is the assembly upon which prior IMGT locus definitions and gene nomenclature have been based. Maintaining consistency with this reference ensures compatibility with existing datasets and allows for direct comparisons across species and individuals without introducing ambiguity due to shifts in chromosome identifiers. However, in analyses and database integration involving the newer T2T assemblies, we adopt the updated chromosome numbers (e.g. IGH on chromosome 15, IGK on 12, IGL on 23). This dual approach preserves continuity with established immunogenetic references while also recognizing the improved structural accuracy of recent assemblies, allowing us to bridge historical annotations with evolving genomic representations.

### Genetic structure and localization of IG loci

In *Pongo abelii*, the chromosome assignments of the IGH, IGL, and IGK loci differ between the Susie_PABv2 assembly and the newer NCBI genome assemblies, whereas in *Pongo pygmaeus* the loci were annotated using only the newer assemblies. These differences reflect updates in chromosome nomenclature and genome assembly rather than biological rearrangements, as discussed in Section 3.1.2. [52, 54].

#### IGH: immunoglobulin heavy chain in *Pongo* species

The IGH locus is located immediately adjacent to the telomere on the long (q) arm in a reverse orientation. For each assembly, the delimitation of the lGH locus was based on the terminal 5′ IGHV gene as well as on the conserved 3′ IMGT borne gene, TMEM121. The IGH locus comprised a total of 167 IGHV genes and 303 alleles in *Pongo abelii* and 177 genes and 263 alleles in *Pongo pygmaeus* were annotated. Distribution of the genes within the IGHV subgroups was not even, similar to that observed in other species. Most of the IGHV genes belong to the IGHV3 subgroup. This subgroup is also the most prevalent in rhesus macaque, gorilla, and human IGH annotation [11, 13, 14, 57]. The other IGHV subgroups were represented by a comparatively low number of genes. Additionally, a difference in gene content among different *Pongo* assemblies was found in and highlighted in Supplementary Fig. S3A and B. Alignment of the functional, open reading frame (ORF) and pseudo in frame IGHV genes, is provided (https://www.imgt.org/IMGTrepertoire/Proteins/proteinDisplays.php?species=Sumatran%20orangutan&latin=Pongo%20abelii&group=IGHV) and (https://www.imgt.org/IMGTrepertoire/Proteins/proteinDisplays.php?species=Bornean%20orangutan&latin=Pongo%20pygmaeus&group=IGHV).

Nine IGHJ genes and 13 alleles in *Pongo abelii* and 9 IGHJ genes and 12 alleles were identified and localized in the *Pongo pygmaeus* IGH locus. The organization of the IGHJ genes is comparable to that of the human J-CLUSTER. They were classified into nine subsets, all named after their human counterparts Supplementary Fig. S3A and B. Nucleotide and amino acid alignment of the functional, ORF, and in-frame pseudogenes IGHJ genes is available (https://www.imgt.org/IMGTrepertoire/Proteins/alleles/index.php?species=Pongo%20abelii&group=IGHJ&gene=IGHJ-overview) (https://www.imgt.org/IMGTrepertoire/Proteins/alleles/index.php?species=Pongo%20pygmaeus&group=IGHJ&gene=IGHJ-overview). The reading frame of the functional IGHJ genes encodes the expected conserved motif WGXG (tryptophan, glycine, any amino acid, and glycine).

Thirty-one IGHD genes belonging to seven sets were identified and localized in both orangutan IGH locus, comprising 35 alleles in *Pongo abelii* and 34 alleles in *Pongo pygmaeus*. Each IGHD set comprises seven genes, except for the IGHD1 set, which is the most represented with six genes, and IGHD7 set, which is the least represented with one gene Supplementary Fig. S3A and B. Alignment of the functional, ORF, and pseudo in frame IGHD genes, is provided (https://www.imgt.org/IMGTrepertoire/Proteins/alleles/index.php?species=Pongo%20abelii&group=IGHD&gene=IGHD-overview) (https://www.imgt.org/IMGTrepertoire/Proteins/alleles/index.php?species=Pongo%20pygmaeus&group=IGHD&gene=IGHD-overview). A 20X zoom view of the D-J-CLUSTER is provided in Supplementary Fig. S4.

Thirteen constant IGHC genes corresponding to 29 alleles encoding the constant region of the H chain isotypes {(IGHM, IGHD, IGHG1, IGHG2, IGHG3 (×3), IGHG4, IGHE (×3), and IGHA (×2)} and associated exons were mapped onto assemblies of *Pongo abelii* with IGHGP1, IGHEP, and IGHG3C only found in reference assembly (GCA_002880775.3). Likewise, 12 constant IGHC genes corresponding to 17 alleles encoding the constant region of the H chain isotypes {(IGHM, IGHD, IGHG1 (×2), IGHG2, IGHG3 (×2), IGHG4, IGHE (×2), and IGHA (×2)} and associated exons were mapped onto assemblies of *Pongo pygmaeus*  Supplementary Fig. S3A and B.

The TMEM121 (transmembrane protein 121) gene, corresponding to NCBI Gene IDs 100451277 in *Pongo abelii* and 129013159 in *Pongo pygmaeus*, is located immediately downstream of the IGH locus at its 5′ end, ~62 kb beyond the terminal constant gene IGHA2 (https://www.imgt.org/IMGTrepertoire/LocusGenes/bornes/bornesIGH.html). In humans and mice, this region is adjacent to the 3′ regulatory region, a key enhancer cluster controlling antibody expression and CSR, suggesting that TMEM121 may be subject to the regulatory architecture of the IGH locus [58].

#### IGL: immunoglobulin lambda chain in *Pongo* species

The IGL is organized into a cluster of variable (IGLV), joining (IGLJ), and constant (IGLC) genes. A total of 105 IGLV genes corresponding to 198 IGLV alleles in *Pongo abelii* and 114 IGLV genes corresponding to 172 IGLV alleles in *Pongo pygmaeus* were annotated. The IGLV genes were classified into 11 subgroups and seven clans defined according to IMGT-ONTOLOGY and to their sequence similarity with the human IGLV subgroups (https://www.imgt.org/IMGTindex/IGLVclans.php). The orangutans IGL J-C-CLUSTER is composed of seven tandems of IGLJ and IGLC genes.

The VPREB1 (V-set pre-B cell surrogate light chain 1, also known as pre-B lymphocyte 1, NCBI Gene ID: 100435541) gene is situated within the immunoglobulin lambda IGL locus, positioned upstream of the lambda variable (IGLV) gene cluster. It encodes a small IG-like protein that partners with λ5 (IGLL1) to form the surrogate light chain component of the pre-B-cell receptor, which plays an essential role in early B-cell differentiation and selection [59].

The locus is flanked by two conserved non-IG “borne” genes (https://www.imgt.org/IMGTrepertoire/LocusGenes/bornes/bornesIGL.html). The 5′ borne gene, TOP3B (DNA topoisomerase III beta) (NCBI Gene IDs: 100434686 in *Pongo abelii* and 129022830 in *Pongo pygmaeus*), lies immediately upstream of the most distal IGLV gene, whereas the 3′ borne gene, RSPH14 (radial spoke head 14 homolog) (NCBI Gene IDs: 100461973 in *Pongo abelii* and 129022797 in *Pongo pygmaeus*), is located downstream of the terminal constant gene IGLC. In humans, TOP3B and RSPH14 are positioned ~43 and 136 kb from the first and last IGL genes, respectively. In orangutans, these borne distances are expanded relative to humans: TOP3B is located 61 kb upstream of the first IGLV gene in *Pongo abelii* and 91 kb in *Pongo pygmaeus*, while RSPH14 resides 128 and 219 kb downstream of the terminal IGLC gene in the same species, respectively.

Within the IGL locus, several region-positioned intervening (RPI) genes including PRAME (NCBI Gene IDs: 100447677 in *Pongo abelii* and 129022829 in *Pongo pygmaeus*), ZNF280A (100440 308 and 129022 790), and ZNF280B (100439940 and 129022789) were also identified. These are non-IG genes integrated within the IGL locus rather than flanking it.

#### IGK: immunoglobulin kappa chain in *Pongo* species

The IGK locus consists of a cluster of variable (IGKV) genes, joining (IGKJ) genes, and a single constant (IGKC) gene. The IGK locus in *Pongo abelii* comprises 79 IGKV genes corresponding to 150 IGKV alleles, 5 IGKJ genes and alleles, and one IGKC gene and allele. The IGK locus in *Pongo pygmaeus* harbours 77 IGKV genes corresponding to 124 IGKV alleles, 5 IGKJ genes corresponding to 6 IGKJ alleles, and one IGKC gene and 2 IGKC alleles.

The IGK locus is flanked by two conserved non-IG “borne” genes: PAX8 (5′) upstream of the most distal IGKV gene and RPIA (3′) downstream of the terminal IGKC gene. In *Pongo abelii*, PAX8 (100189903) is located 312 kb upstream and RPIA (100447020) 136 kb downstream of the IGK gene cluster, whereas in *Pongo pygmaeus*, PAX8 (129030568) lies 260 kb upstream and RPIA (129025031) 132 kb downstream of the corresponding IGK region (https://www.imgt.org/IMGTrepertoire/LocusGenes/bornes/bornesIGK.html).

### IG loci structure and gene functionality across five *Pongo* assemblies

To investigate further the structural diversity and gene composition of the IGH locus across the assemblies studied, we analyzed key characteristics, such as number of genes and gene functionality Supplementary Tables S2–S4. Figure 1 provides an overview of the gene composition within complete IG loci across diverse *Pongo* assemblies. The gene composition and functionality classification of the complete IG loci, IGK (A), IGL (B), and IGH were examined across five chromosome-localized *Pongo* assemblies.

Each locus was analyzed according to IMGT gene functionality categories, including functional, ORF, and pseudogene classes. The stacked horizontal bars illustrate the proportional distribution of each functionality type per assembly and per locus, while the accompanying pie charts summarize the functionality proportion of variable genes across assemblies.

Across all loci, pseudogenes constituted the largest proportion of variable genes, most prominently in the IGL locus where they accounted for ~70% of the repertoire. The IGH locus (Fig. 1C) was distinguished primarily by its overall expansion relative to the light chain loci (IGK and IGL) (Fig. 1A and B). In comparison, the IGK locus contained a lower proportion of pseudogenes (52%) and exhibited the highest proportion of functional genes (42%), whereas functional genes represented only 28%–33% of the IGH and IGL repertoires. ORFs contributed only a minor fraction across all loci (2%–6%). In contrast to the variability observed in the V gene repertoire, the J, D, and C regions were comparatively conserved across genomes, showing little or no variation and indicating strong selective maintenance of their structural and functional integrity.

The consistent scale across panels highlights clear differences in locus size, with the IGH locus being substantially larger and functionally more diverse than the light chain loci, reflecting an expanded heavy chain gene repertoire relative to the more compact IGK and IGL loci.

**Figure 1.**
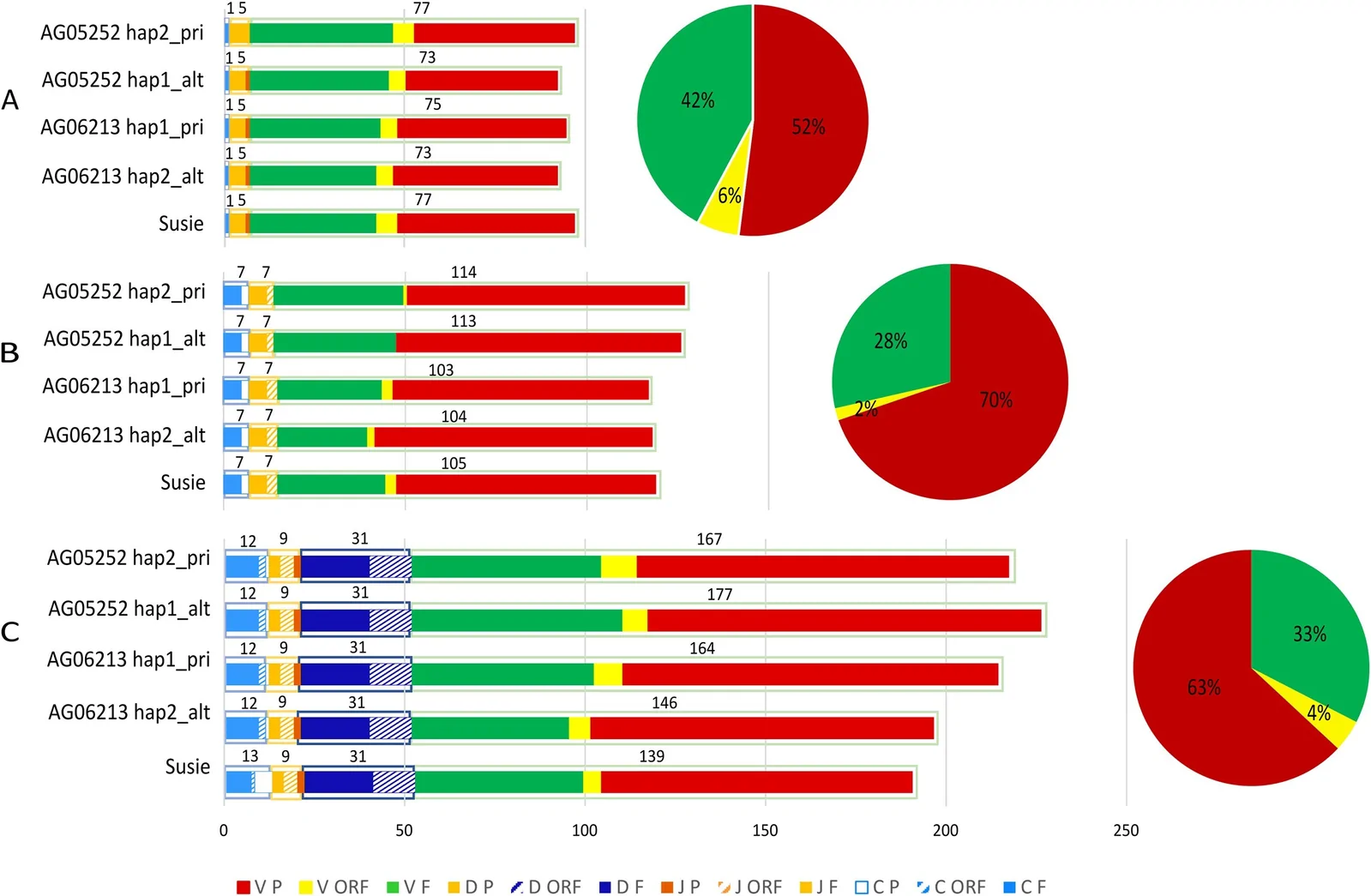
Gene composition and functionality classification of complete IG Loci IGK (**A**), IGL (**B**), and IGH (**C**) within five chromosome-localized *Pongo* assemblies. This graph displays the gene count and functionality distribution for each gene type (variable, diversity, joining, and constant), with colors applied according to the IMGT color menu, reflecting the IMGT functionality per gene type. All loci are plotted on the same genomic scale, enabling direct comparison of locus length, gene number, and functional diversity. Pie charts on the right display the proportion of variable genes per functionality for each locus.

### Structural variations/haplotype-specific insertions and duplications

The high frequency of structural variation observed across the IGHV locus is thought to be driven by its repetitive nature, largely caused by the duplication of IGHV genes [60]. To explore this structural complexity in detail, holistic maps of the immunoglobulin heavy chain (IGH) locus in *Pongo abelii* and *Pongo pygmaeus* were constructed from localized genome assemblies to illustrate the overall organization and structural variation within the locus (Supplementary Fig. S3A and B). The locus shows distinct shaded regions, each representing a structurally or functionally unique segment characterized by haplotype-specific insertions or deletions. The boundaries and composition of each region were validated through comparison across multiple haplotypes, assemblies, and individuals from both *Pongo abelii* and *Pongo pygmaeus*, and were further supported by ultra-long-read alignments visualized in IGV [61]. Continuous long-read coverage spanning the variant regions confirmed that the observed differences were supported by the underlying sequencing data and were not attributable to assembly artifacts (Supplementary Figs S6–S11).

Across the analyzed genomes, the telomeric end of the IGH locus in *Pongo* species shows a high degree of conservation with the human and gorilla IGHV gene cluster. In *Homo sapiens, Gorilla gorilla gorilla, Pongo abelii* (AG06213), and *Pongo pygmaeus*, this region contains a series of IGHV genes belonging to the same IGHV subgroups, exhibiting similar functionality. In the human and gorilla, this telomeric cluster includes genes such as IGHV5-78, IGHV(II)-78–1, IGHV3-79, IGHV4-80, IGHV7-81, and IGHV(III)-82, which are also represented in *Pongo abellii* (AG06213) and *Pongo pygmaeus* with conserved order and orientation. The gene content and order are remarkably conserved, indicating a shared ancestral organization of the telomeric IGHV domain among great apes. In contrast, the non-T2T Susie assembly terminates at IGHV(III)-139 and lacks the most distal telomeric IGHV genes; however, these genes are present in the more recent Susie assemblies, indicating that their absence is likely attributable to incomplete assembly of the telomeric region Supplementary Fig. S5.

Regions 2 and 3 include the genes IGHV3-134 and IGHV3-123/IGHV3-122, which are present in some *Pongo* assemblies but absent in others (Supplementary Fig. S5). This variable presence suggests a small-scale structural polymorphism within this region.

Region 4 contains a dense cluster of IGHV genes between genes IGHV3-115 and IGHV1-119. The number of genes in this region differ among the *Pongo abelii* assemblies, indicating structural diversity within this region (Supplementary Fig. S5).

Region 5 is located between IGHV1-98 and IGHV4-104 and consists of tandem repeats of a five-gene block. The first block spans IGHV4-99 through IGHV1-103, and this same five-gene block is duplicated multiple times. These duplications give rise to IGHV4-103-1 through IGHV4-103-15, representing three consecutive repetitions of the same five-gene structure, each retaining the same IGHV subgroup composition. The number of repeated blocks is not constant and varies across assemblies. In the NHGRI_mPonPyg2-v2.0_pri (primary haplotype) (Fig. 2C), only a single instance of this block is present, spanning IGHV4-103-1 through IGHV4-103-5. In contrast, the NHGRI_mPonPyg2-v2.0_alt (alternate haplotype) (Fig. 2A) shows a much larger expansion, extending continuously to IGHV4-103-15, corresponding to three full consecutive copies of the same five-gene structure. Similarly, the *Pongo abelii* T2T assemblies (NHGRI_mPonAbe1-v2.1_pri and NHGRI_mPonAbe1-v2.1_alt) each contain a single copy of the duplicated block, resembling the organization observed in NHGRI_mPonPyg2-v2.0_pri.

**Figure 2.**
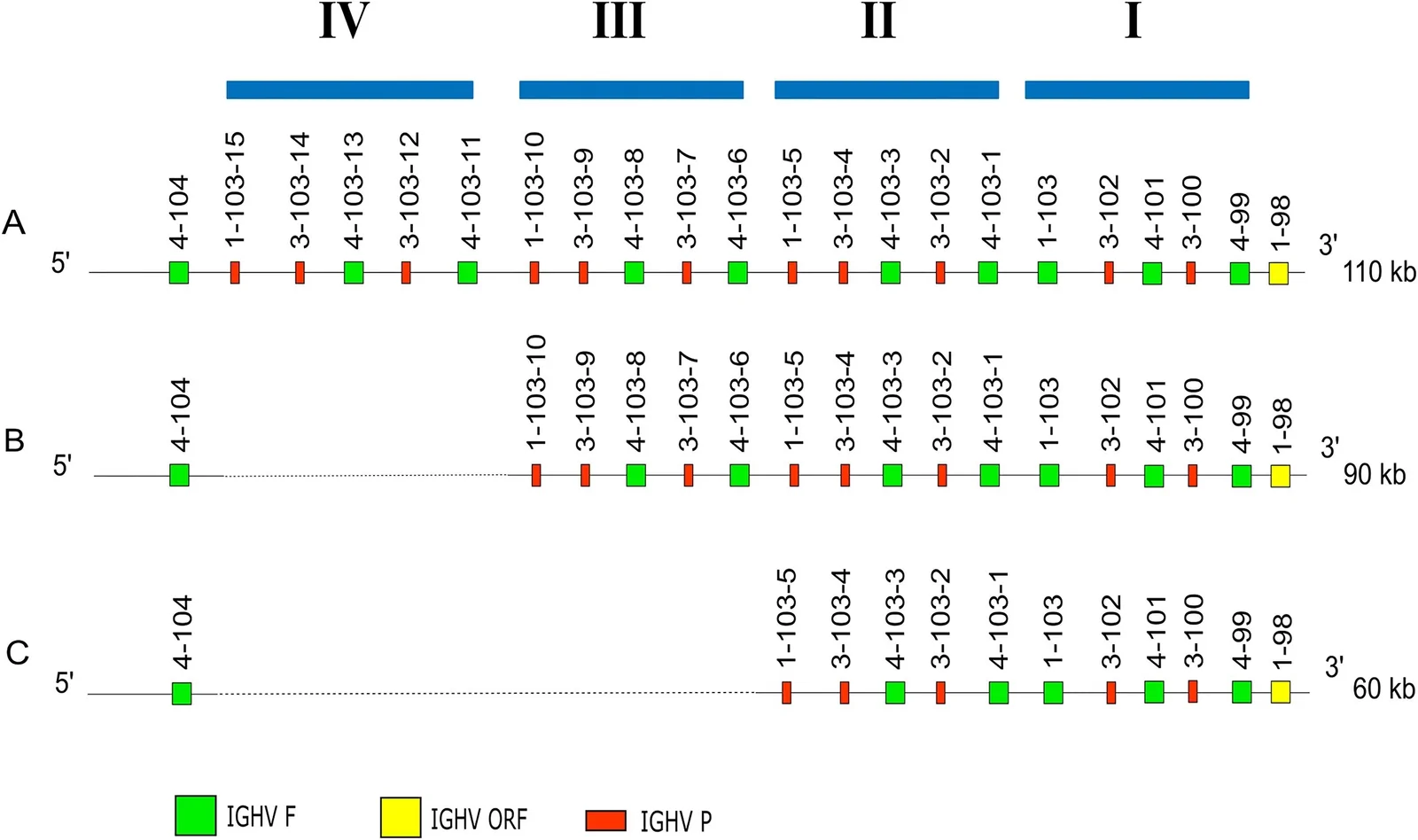
Haplotype-specific expansion of a duplicated IGHV block in Region 5. Region 5, located between IGHV1-98 and IGHV4-104, contains tandem repeats of a five-gene block that runs from IGHV4-99 through IGHV1-103. This same block is duplicated in series, giving rise to IGHV4-103-1 through IGHV4-103-15, corresponding to up to three consecutive copies with identical IGHV subgroup composition. The number of block copies varies across *Pongo* assemblies.

In the Susie_PABv2, this region was not fully represented, most likely due to its highly repetitive structure, which makes it difficult to resolve completely using conventional assembly methods. Analysis of the new Susie haplotype-resolved assemblies (Susie_PAB_hifiasm-v0.15.2.pri and Susie_PAB_hifiasm-v0.15.2.alt) provided additional resolution of this area: the primary haplotype contains genes IGHV4-103-1 to IGHV4-103-5 (Fig. 2C), whereas the alternate haplotype extends up to IGHV4-103-10 (Fig. 2B). These newer assemblies therefore complement the Susie_PABv2 reference by filling previously unresolved repetitive regions. Supplementary Figs S7–S10 demonstrate continuous long-read support across this duplicated region, characterized by uniform read alignment and the absence of coverage gaps or split mappings.

Regions 6 and 7 encompass the constant region (IGHC) genes of the IGH locus. Among the *Pongo abelii* assemblies analyzed in this study, this region generally contains the expected IGHC repertoire, including IGHGP, IGHG2, IGHG3, IGHE, and IGHA, arranged in the canonical order and orientation. However, in the Susie_PABv2 assembly, three additional IGHC genes IGHGP1, IGHEP, and IGHG3C were annotated, while IGHGP and IGHG2 were absent.

Read mapping across this region (Supplementary Fig. S10) showed clear coverage dropouts and low mapping quality precisely at the positions corresponding to IGHGP and IGHG2, suggesting these absences result from unresolved N-gap, reflecting incomplete sequence representation rather than true gene loss. Conversely, the regions annotated as IGHGP1, IGHEP, and IGHG3C coincide with irregular coverage patterns, multiple secondary alignments, and local sequence gaps, indicative of false duplications or misassembled fragments in low-complexity constant-region sequences.

Further confirmation came from the recently generated Susie_PAB_hifiasm-v0.15.2.pri and Susie_PAB_hifiasm-v0.15.2.alt assemblies of the same individual, which recovered IGHGP and IGHG2 but did not contain IGHGP1, IGHEP, or IGHG3C. Although these assemblies are not included in public databases due to not meeting IMGT quality criteria (Supplementary Table S1), their concordance with other *Pongo abelii* and *Pongo pygmaeus* individuals confirms that the three additional IGHC genes in Susie_PABv2 are assembly artifacts (Supplementary Fig. S10). Accordingly, IGHGP1, IGHEP, and IGHG3C will be flagged as false gene predictions in our dataset. Taken together, Regions 5–6 show that the constant region is generally conserved across orangutan genomes, and the apparent differences in Susie_PABv2 reflect assembly-specific anomalies rather than genuine structural variation.

### Haplotype-resolved allelic variation of immunoglobulin variable gene families in analyzed *Pongo* assemblies

To further characterize structural variation within the IG loci, we examined genes present exclusively on one haplotype, distinguishing between haplotype-specific gene deletions and haplotype-specific insertions or duplications. Haplotype-specific genes, which are present on one haplotype but completely absent on the homologous chromosome, typically reflect deletion events and result in reduced copy number. In contrast, we also identified instances where additional gene copies were found on only one haplotype, with no corresponding ortholog on the other.

Across all three IG loci, the distribution of identical alleles, allelic variants, and haplotype-specific genes revealed pronounced haplotype-level diversity. The majority of genes were classified as allelic variants, indicating that sequence variation between shared genes is more common than gene gain or loss. Notably, the IGH locus contained the highest proportion of haplotype-specific genes in both *Pongo pygmaeus* and *Pongo abelii* assemblies, suggesting that this region undergoes frequent structural variation, including haplotype-specific deletions and insertions. In contrast, the IGLV and IGKV loci were dominated by allelic variants but showed largely balanced gene content between haplotypes, consistent with stronger sequence conservation and lower rates of copy-number turnover. Comparative analysis between the two species further showed that the *P. abelii* dataset exhibits slightly higher overall gene-level allelic variation across all three loci, indicating greater allelic diversification within the analyzed IG repertoires. Together, these results highlight extensive allelic and structural heterogeneity within orangutan IG loci, with the IGH locus emerging as a hotspot for haplotype-specific structural variation (Fig. 3).

**Figure 3.**
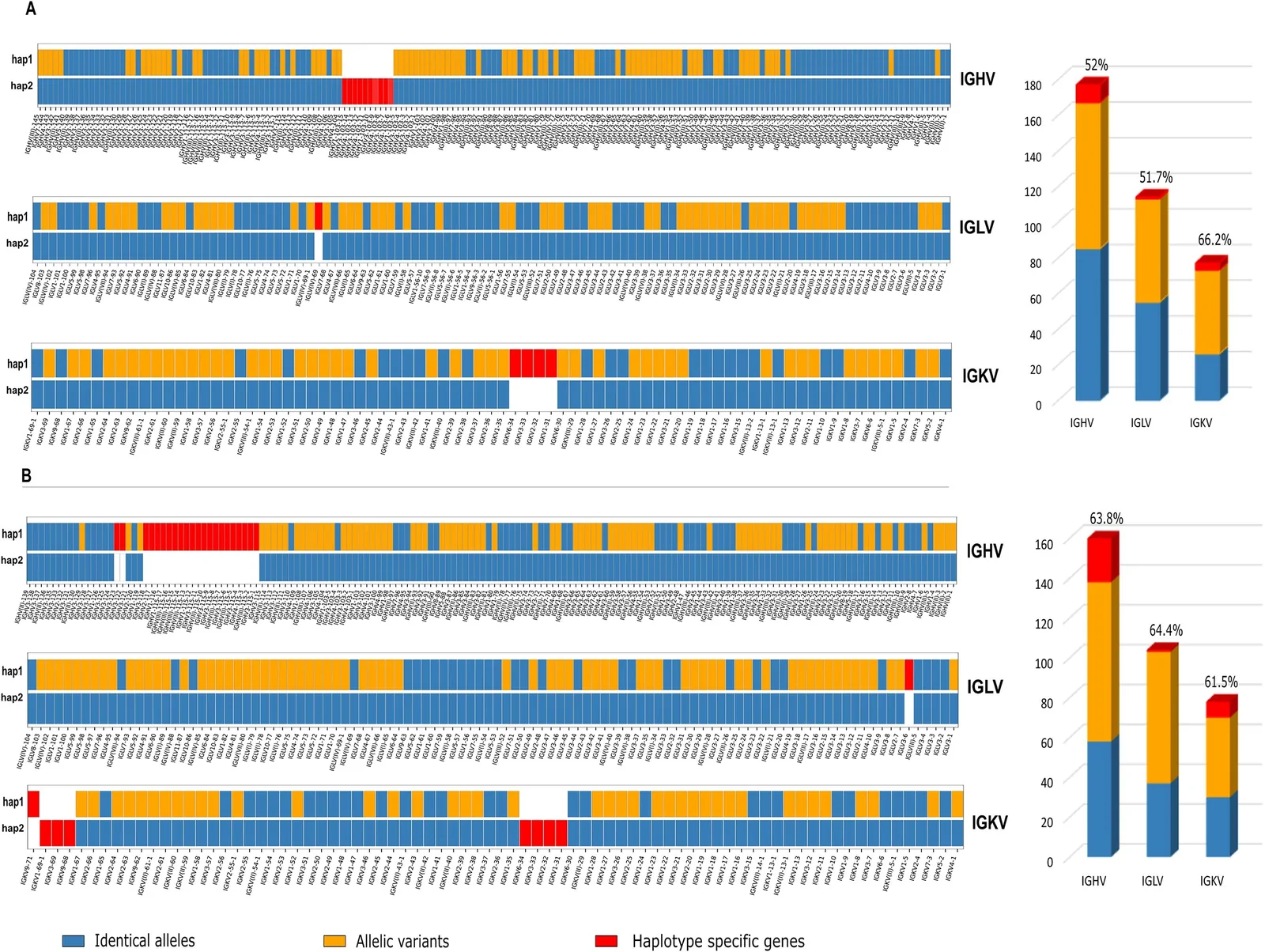
Haplotype-resolved allelic variation across IG variable loci *Pongo* genome assemblies. Panels (**A**) and (**B**) show comparisons of *Pongo pygmaeus* and *Pongo abelii* haplotypes, respectively, for the immunoglobulin heavy (IGHV), lambda (IGLV), and kappa (IGKV) variable gene families. Each horizontal track represents one haplotype (hap1 and hap2) aligned gene-by-gene. The colors indicate the degree of allelic divergence at each gene locus: blue denotes identical alleles present in both haplotypes, orange denotes allelic variants present on both haplotypes but differing in sequence, and red denotes haplotype-specific genes present on only one haplotype. Bar charts to the right of each panel summarize the proportions of the three categories for each locus, with percentage values representing overall gene-level allelic variation between the two phased haplotypes of the same individual rather than population-level heterozygosity. The figure illustrates that analyzed orangutan assemblies exhibit substantial allelic and structural variation across the IGHV, IGLV, and IGKV loci, with *P. abelii* showing slightly higher overall allelic variation than *P. pygmaeus*. Assemblies used: NHGRI_mPonPyg2-v2.- (pri/alt) and NHGRI_mPonAbe1-v2.- (pri/alt).

### Switch sequence analysis in IGHC genes

To investigate the regulatory architecture of orangutan IGHC genes, we first extracted the 5-kb strand-aware upstream switch-region sequences for each constant gene from the *Pongo pygmaeus* and *Pongo abelii* reference genomes (GCF_028885625.2 and GCF_028885655.2, respectively). We quantified the density of canonical AID hotspot motifs, including GAGCW (W = A or T) and GGGGBT (B = C, G, or T), and calculated the GC content across the same regions (Supplementary Table S5). When visualized as bar charts, these features showed a highly concordant and gene-specific enrichment pattern between the two species, indicating strong conservation of switch-region architecture across orangutan assemblies (Fig. 4). Quality control plots showing sliding-window distributions of motif density and GC fraction were generated for each region to verify extraction accuracy and to visualize local enrichment patterns in both *Pongo* species Supplementary Fig. S12.

**Figure 4.**
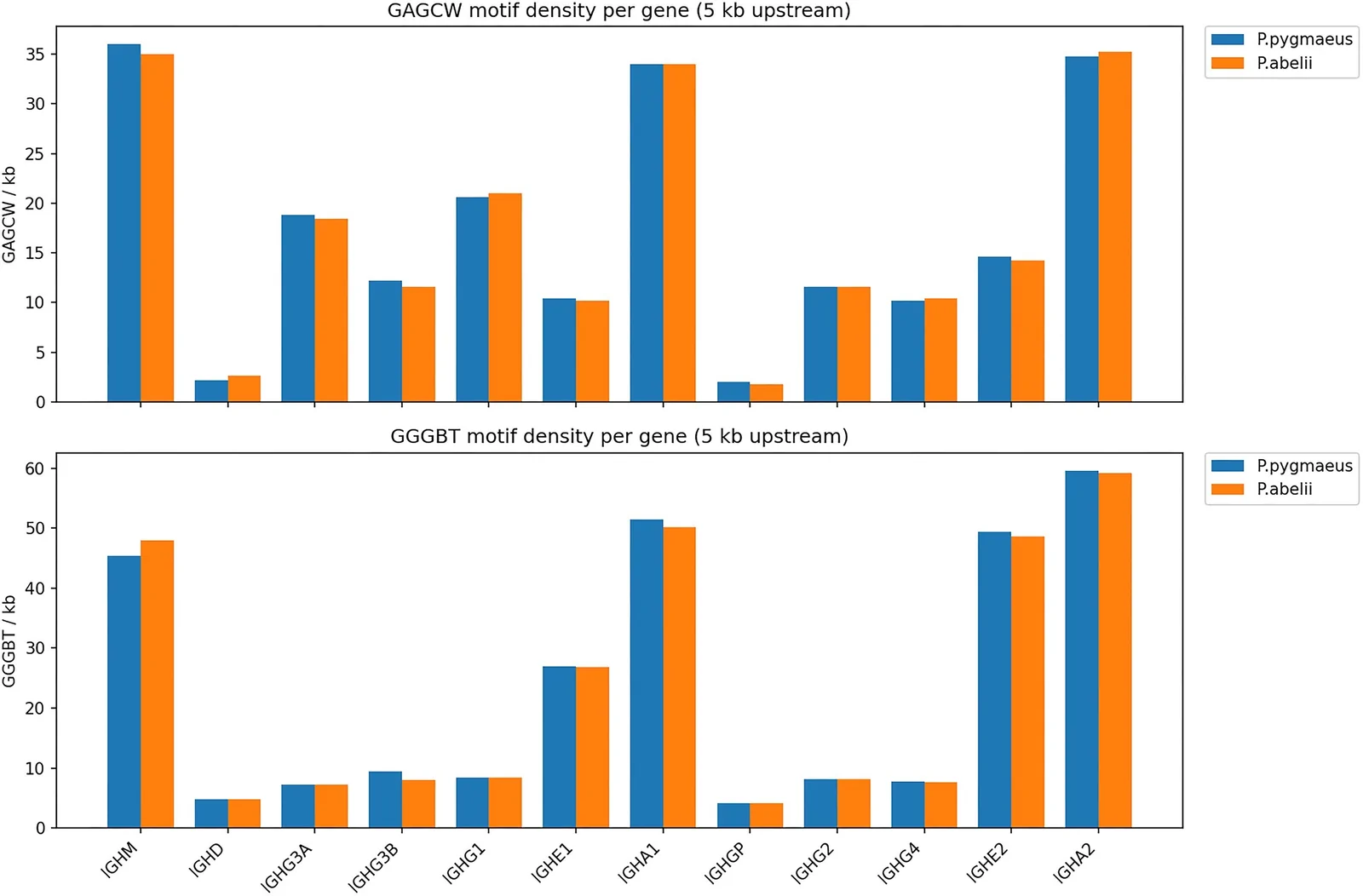
Density of canonical AID hotspot motifs in the 5-kb upstream promoter region of each IGH constant-region gene in *Pongo pygmaeus* and *Pongo abelii*. Each bar represents the number of predicted AID target motifs per kilobase immediately upstream of the indicated IGH constant gene. The top panel shows the density of GAGCW motifs, and the bottom panel shows GGGGBT motifs. Bars represent *P. pygmaeus* and *P. abelii*, as indicated in the figure legend. Although absolute densities vary across constant-region subclasses, both species show a broadly conserved ranking with notably high motif enrichment upstream of IGHA1, IGHE, and IGHG2/IGHG3, and minimal enrichment at IGHD and IGHG3/IGHG4.

To further explore the structural organization underlying this conservation, we generated Gepard self-alignment dotplots for *Pongo abelii*, both across the entire IGHC constant-gene cluster and individually for each upstream switch-region (Fig. 5) [46].

**Figure 5.**
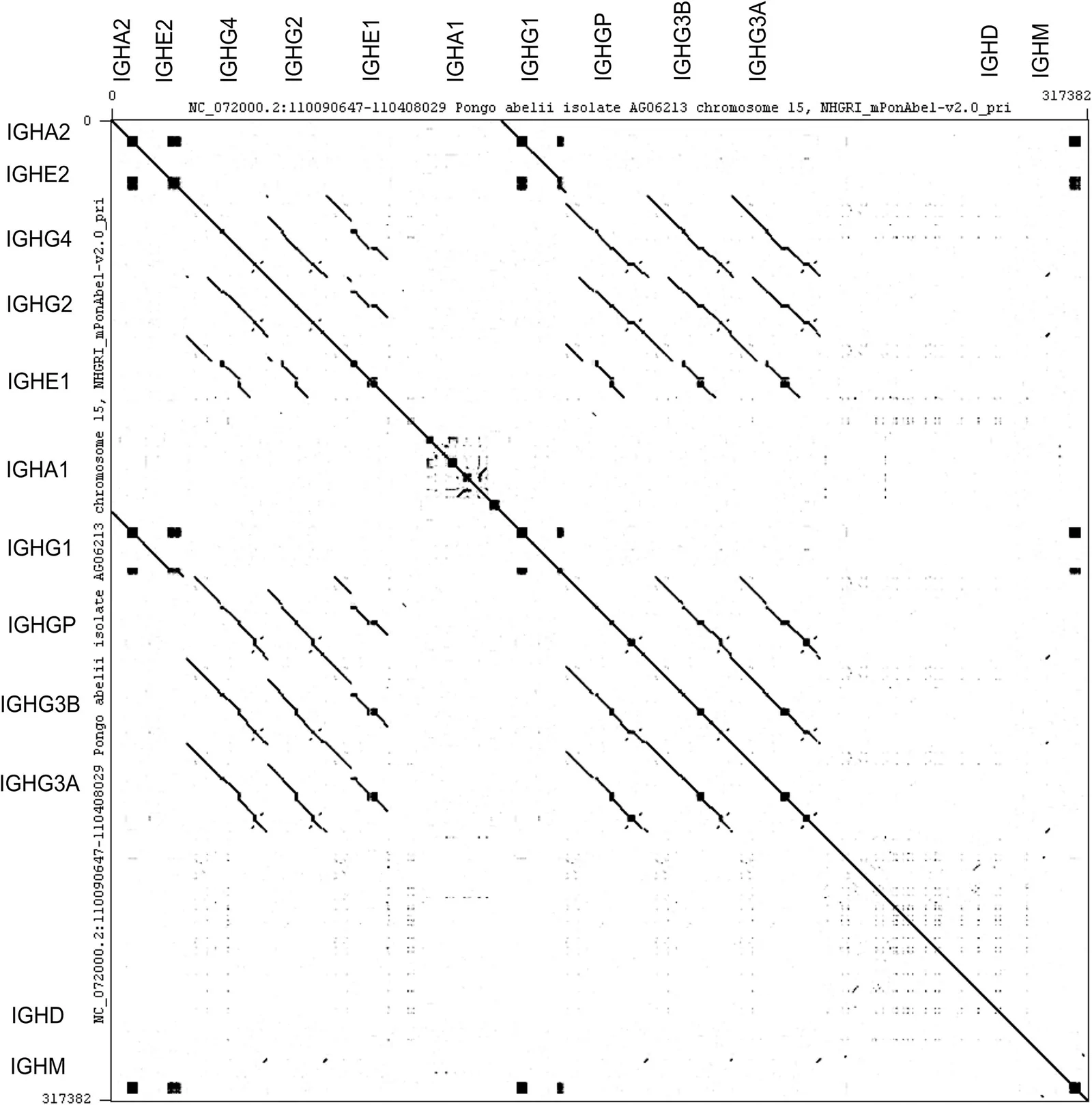
Dotplot of the IGHC locus in *Pongo abelii*. Self-comparison of the constant region cluster generated with standalone Gepard application [46] shows extended tandem repeats upstream of IGHM and IGHA genes, consistent with classical switch regions. IGHE and IGHG genes display shorter or fragmented repeat signals, while IGHD and IGHGP lack detectable repeats. We used a word size of 14 because it is sufficiently small to detect the short, high-identity repeats characteristic of IG switch regions, while still suppressing background noise from low-complexity sequences.

Distinct patterns were observed among genes corresponding to their known switch-region strength Supplementary Fig. S13. The IGHM, IGHA1, and IGHA2 upstream regions displayed dense, continuous diagonals indicative of extensive tandem repeats, consistent with the canonical Sμ and Sα regions. In contrast, the IGHG and IGHE genes showed shorter or fragmented repeat tracts, reflecting more heterogeneous and less extensive switch regions. The upstream regions of IGHG2, IGHG3B, and IGHG4 also exhibited short inverted diagonals, indicating the presence of localized inverted repeat sequences. The IGHD and IGHGP upstream regions lacked internal homology signals beyond the self-identity diagonal, suggesting the absence of switch-like repeats. Overall, these per-gene dotplots confirmed the patterns seen at the cluster level (Fig. 5), demonstrating strong repeat enrichment upstream of μ and α genes, intermediate organization in γ and ε, and no discernible switch structures in δ or GP.

### Analysis of recombination signal sequences

Sequence logo analysis of RSSs from functional IG V, D, and J genes revealed the expected conserved motifs characteristic of RAG-mediated recombination (Fig. 6). Across all three loci (IGH, IGK, and IGL), the canonical heptamer (CACAGTG) and nonamer (ACAAAAACC) motifs were strongly conserved, consistent with previous reports [5, 13, 62–64]. The heptamer showed nearly invariant conservation at positions 1–7, particularly the central CACAGTG core, while the nonamer displayed high A content. The first three bases (CAC) of the heptamer and the central A-rich core of the nonamer are critical for RAG1/2 recognition and cleavage during V(D)J recombination. Minor locus-specific variations were observed at the flanking nucleotides and within the spacer regions, suggesting subtle differences in recombination efficiency or RAG binding preferences. The spacer length distribution confirmed the expected predominance of 12 and 23-bp spacers, validating the integrity of the extracted sequences. Together, these results confirm that the RSS motifs across functional IG genes retain the canonical sequence features necessary for efficient V(D)J recombination. Figure 6 shows the consensus RSS motifs in *Pongo abelii*. Comparison of the orangutan RSS consensus sequences with the corresponding human functional RSS consensus sequences was performed using the IMGT Consensus Tool. Identical heptamer and nonamer consensus motifs were observed across the IGH, IGK, and IGL loci, indicating no species-specific consensus differences expected to affect V(D)J recombination efficiency.

**Figure 6.**
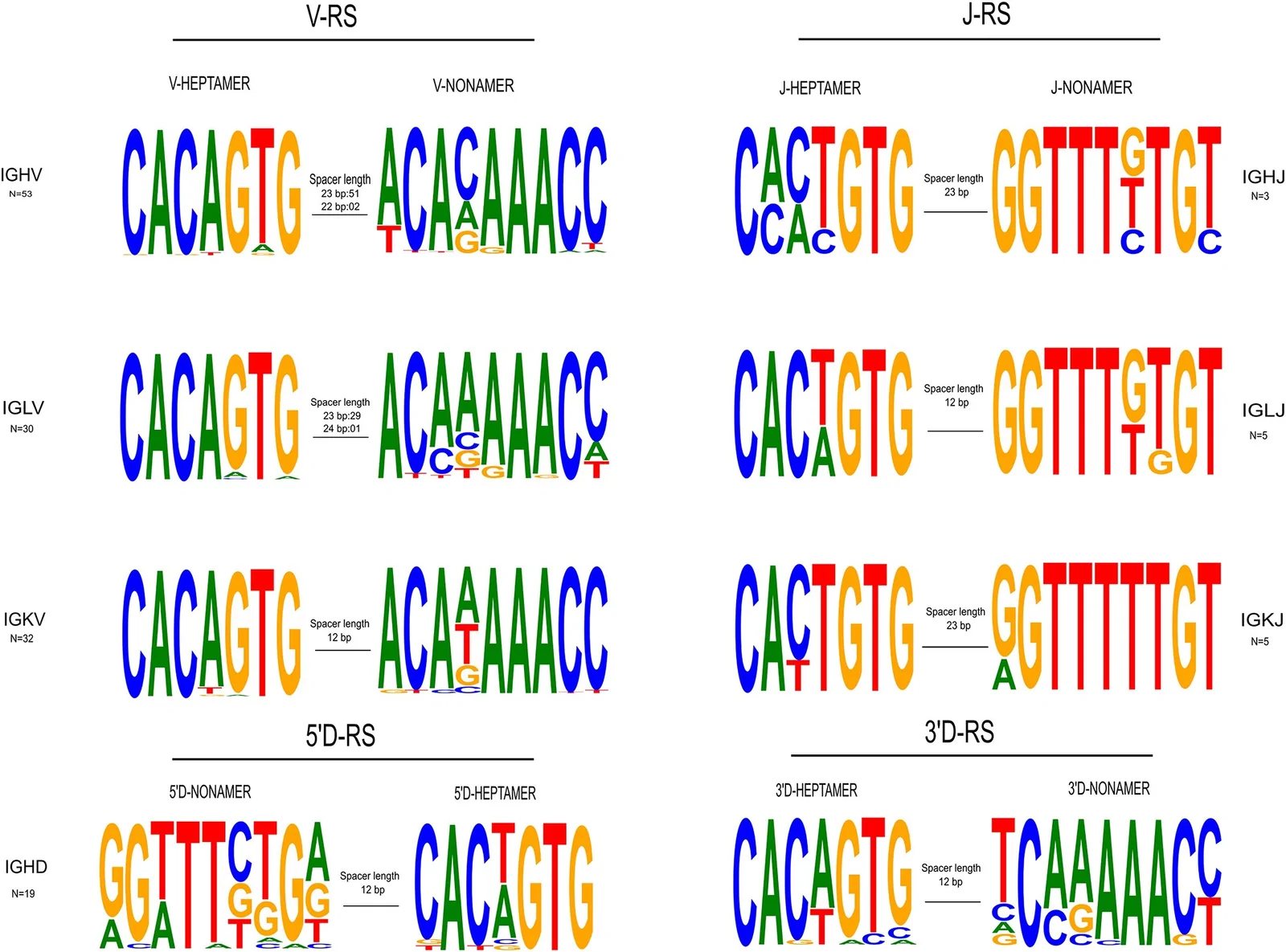
Consensus motifs of RSSs from functional IG genes in *Pongo abelii*. Sequence logos derived from functional IG V, D, and J genes highlight the highly conserved heptamer (CACAGTG) and nonamer (ACAAAAACC) motifs, which flank coding segments and direct RAG-mediated recombination. Both motifs show strong positional conservation, particularly within the core CAC and ACA elements, consistent with their essential role in synapsis and cleavage. The observed spacer length distribution confirmed the expected 12/23-bp rule, with minor locus-specific sequence variability suggesting potential fine-tuning of recombination efficiency across loci.

To quantitatively compare RSS conservation among the IGH, IGK, and IGL loci, the percentage identity of each RSS sequence relative to its corresponding locus-specific consensus sequence was calculated (Supplementary Table S7 and Supplementary Fig. S16). No significant differences in sequence conservation were detected among loci for the V-heptamer (Kruskal–Wallis, *P* = .941), J-heptamer (*P* = .061), V-nonamer (*P* = .131), or J-nonamer (*P* = .557). Likewise, none of the pairwise comparisons remained significant after Benjamini–Hochberg correction (Supplementary Tables S7–S9).

### Phylogeny of IG V genes

Across genome assemblies of *Homo sapiens, Pongo abelii, Pongo pygmaeus*, and *Gorilla gorilla gorilla*, we quantified the immunoglobulin heavy-chain (IGHV) and light-chain (IGKV, IGLV) V-CLUSTERS by taking into account the total V-CLUSTER length and the number of annotated V genes (Supplementary Table S6).

The mean gene density values revealed clear locus- and species-specific patterns. Among all four primates, the IGHV locus showed the greatest divergence, with *Homo sapiens* exhibiting the highest density, indicating a more compact and gene-rich heavy-chain V-CLUSTER relative to the other species. In contrast, *Gorilla gorilla gorilla* displayed a noticeably lower IGHV density, reflecting a more expanded but less gene-dense organization of this locus Fig. 7A.

**Figure 7.**
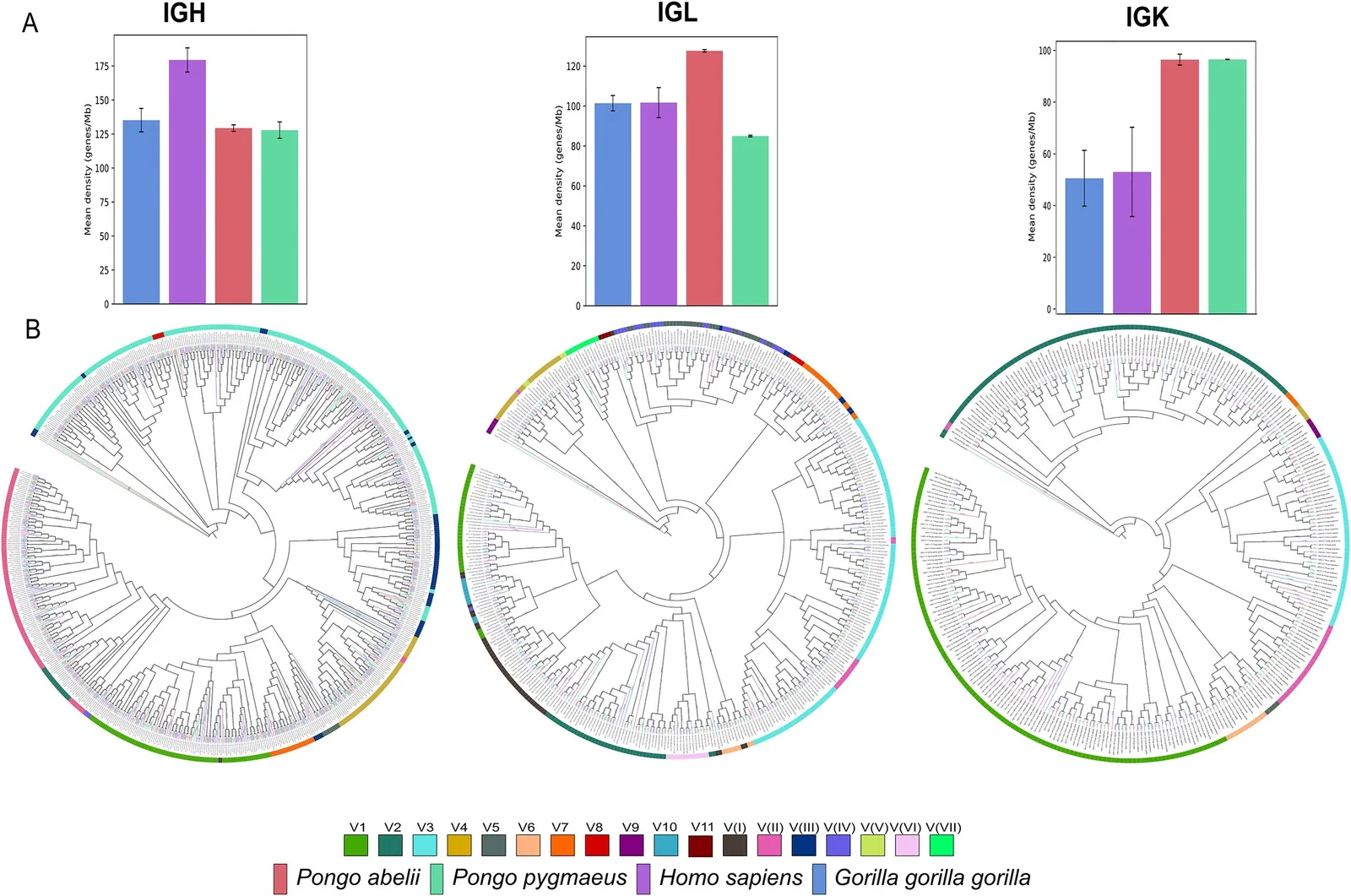
Comparative analysis and phylogenetic relationships of IG variable (V) genes across three loci in great apes. (**A**) Gene density distributions (genes/Mb) for the IGHV, IGKV, and IGLV loci in *Homo sapiens, Pongo abelii, Pongo pygmaeus*, and *Gorilla gorilla gorilla*. Each bar represents the mean gene density ± SD across genome assemblies, showing interspecies variation in V-gene organization. (**B**) Maximum likelihood phylogenies of IGHV, IGKV, and IGLV V genes, color-coded by species and subgroup. Phylogenetic clustering reveals conserved subgroup structures with lineage-specific expansions and species-specific divergence patterns across loci.

The IGKV locus consistently showed the lowest density across all species, suggesting that this light-chain locus is generally more dispersed. However, *Pongo abelii* showed the highest density within IGKV, indicating a relatively compact organization specific to this orangutan lineage (Fig. 7A).

For the IGLV locus, *Pongo abelii* again demonstrated the highest density, in contrast to *Homo sapiens*, which showed a more moderate density, suggesting that orangutans may favor a more compact light-chain organization, whereas humans prioritize a denser heavy-chain locus instead (Fig. 7A).

These trends indicate that locus compaction is not uniform across primate species and that heavy- and light-chain loci follow distinct evolutionary trajectories, with *Homo sapiens* optimizing IGHV density, while orangutans favor higher density in light-chain loci, particularly IGLV.

The relationship between V-CLUSTER size and annotated V-gene count across individual genome assemblies is shown in Supplementary Fig. S14, illustrating the assembly-level variation underlying the mean gene density values presented in Fig. 7A.

To explore the evolutionary relationships of IG genes across primates, we conducted a comparative analysis focusing on the 01 alleles of IGHV genes shared among *Pongo abelii, Pongo pygmaeus, Homo sapiens*, and *Gorilla gorilla gorilla*. Multiple sequence alignments and phylogenetic tree reconstructions were performed to assess sequence divergence and infer evolutionary relationships within and across these subgroups. The resulting phylogeny revealed clear clustering by IGV subgroups, with orthologous genes from different species grouping closely together, reflecting a high degree of evolutionary conservation. Phylogenetic reconstruction of all annotated V genes (Fig. 7B) showed conserved subgroup architectures across species, with clear clustering of orthologous genes and distinct species-specific clades. The IMGT clan reference sequences are consistently clustered within their respective subgroups, confirming accurate phylogenetic placement and evolutionary correspondence across loci. IMGT clans represent ancient evolutionary lineages of IG variable (V) genes that share conserved framework motifs and structural features. Their inclusion provides an evolutionary reference framework, ensuring accurate subgroup classification and enabling consistent cross-species comparisons of V-gene diversification.

## Discussion

Advances in large-scale DNA sequencing and genome assembly have greatly improved the characterization of complex immune loci in nonhuman primates. However, comprehensive analyses of IG loci across the genus *Pongo* remain limited, largely due to the challenges associated with obtaining high-quality genomic resources from critically endangered great apes. In this study, five genome assemblies representing two *Pongo abelii* individuals and two assemblies corresponding to a single *Pongo pygmaeus* individual were included. Currently, no genome assembly is available for *P. tapanuliensis*, which restricts direct comparisons with this recently described species. As additional genomic data become accessible, particularly for *P. tapanuliensis*, a more complete understanding of species-specific diversity and interspecies differences within the *Pongo* genus is expected to develop. Furthermore, the IMGT-standardized annotation framework established in this study will facilitate the rapid and consistent annotation of IG loci in future *P. tapanuliensis* genome assemblies, enabling direct comparative analyses across all extant orangutan species.

### Structural variation and haplotype diversity in the orangutan IGHV repertoire

Segmental duplications have contributed significantly to the expansion of various protein-coding gene families, often through repeated duplication events of specific sequences [65–67]. The immune loci exhibit more rapid divergence and large structural differences between haplotypes and species than many other genomic regions [68]. Consistent with this pattern, duplications and deletions are widespread throughout the orangutan IGHV gene cluster. The repeating IGHV modules identified in this study (e.g. the five-gene blocks in Region 5), together with haplotype-specific gains and losses and allelic variation in gene functional status, are concordant with models of duplication-mediated structural evolution [69–73]. The organization of Region 5, where a five-gene IGHV block containing two functional and three pseudogene copies is duplicated in tandem, closely resembles the structural pattern reported by [74], who described a two-functional and three-nonfunctional IGHV block duplication in the human lineage. The presence of this duplicated architecture in both *Pongo* and *Homo* suggests that it predates the human-orangutan split and likely represents an ancestral feature of great ape IGHV locus evolution. The heterozygosity observed across the IGH locus further supports the dynamic nature of this region, where copy number variation, gene gain and loss, and allelic diversification contribute to haplotype-specific repertoire diversity.

### Conservation and diversity of IGH constant genes and switch sequences

In the present study, we found that the IGHD gene is conserved as an ORF across all examined orangutan genomes. Initial sequence analysis suggested that the gene was disrupted by frameshifts within the CH1 exon; however, detailed re-examination showed that the observed sequence differences result from two closely spaced insertion events with a combined length of six nucleotides. These insertions locally reorganize the codons within the DE turn, preserve the downstream coding frame, and result in a net addition of two amino acids compared with the human IGHD sequence. Notably, the insertions occur within the DE turn, a loop region of the IG constant domain recognized by the IMGT unique numbering system as having variable length, allowing insertions to be accommodated without disrupting the overall domain architecture. Interestingly, *Pongo* species are the only primates currently annotated in IMGT to exhibit a DE turn of this length (https://www.imgt.org/IMGTrepertoire/Proteins/). The affected region also exhibits local amino acid substitutions and insertions while maintaining the overall coding potential of the gene (Supplementary Fig. S15). Consequently, IGHD was reclassified as an ORF rather than a pseudogene. However, functional classification cannot be established from genomic sequence alone in the absence of expression or protein-level evidence.

In humans, IgD is an antibody present at very low concentrations in serum; nevertheless, it is widely expressed on the surface of B cells as part of the B cell receptor complex in many species [75–78]. Knowledge at the genetic level of IgD in species other than human and rodents has only begun to accumulate recently, and has demonstrated that IGHD is widespread throughout vertebrates and is extremely diverse [78]. However, unlike humans and other great apes, the IGHD gene in all examined orangutan haplotypes contains a localized 6-nt in-frame insertion within the CH1 exon, resulting in local amino acid changes while preserving the overall coding frame. Whether these sequence differences influence IgD expression or biological function in orangutans remains to be determined.

When compared with the sequences described in humans by Mills *et al*. [45], the orangutan switch regions show strikingly similar features. In both species, Sμ and Sα regions are enriched in tandem pentamer repeats and display high GC content, whereas Sγ and Sε regions contain shorter, more fragmented repeats with lower motif density. The absence of a distinct switch-like region upstream of IGHD is consistent between orangutan and human, and the orangutan IGHGP likewise lacks canonical switch motifs. Thus, while the exact repeat structure and motif composition may differ, the relative distribution of strong and weak switch regions across the constant gene cluster is comparable between orangutan and human. In most mammals, expression of IGHD is generated primarily through alternative RNA splicing of a shared IGHM-IGHD primary transcript, rather than by classical CSR [79]. Consequently, the switch region (Sδ) upstream of IGHD is generally weak or poorly defined in primates compared to the canonical Sμ, Sγ, Sα, and Sε elements [68]. The absence of a strong Sδ region in orangutan assemblies is therefore consistent with the broader mammalian pattern, where CSR to IGHD is rare or nonfunctional.

Previous studies have shown that IGHG copy number is highly variable across primates, particularly between New World monkeys and apes, indicating that IgG evolution has proceeded in lineage-specific ways. In *Pongo* species, we annotated 6 IGHG genes: IGHG3A, IGHG3B, IGHG1, IGHG2, IGHG4, and IGHGP. Among these, IGHG3A, IGHG1, IGHG4, and IGHGP appear to be functional, while IGHG3B and IGHG2 are ORFs. In humans, copy number variation is documented at the IGHG locus, particularly affecting IGHG4, which can occur in more than one copy in certain haplotypes [e.g. IGHG4A / duplicated IGHG4 (IGHG4A)]. Therefore, the total number of IGHG genes in humans is not fixed, and although the modal or reference haplotype is often described with five IGHG genes, some individuals may carry six IGHG genes including the duplicated IGHG4 (IGHG4A) [14]. The coexistence of four functional IGHG genes together with two ORF copies is consistent with a birth-and-death mode of evolution, as described for primate IgG genes by Garzón-Ospina and Buitrago [80], while the retention of orthologs shared with other great apes indicates that divergent evolution also contributes to the maintenance and functional specialization of conserved IGHG lineages.

### Organization of immunoglobulin light chain loci (IGK and IGL)

The organization of the IGK and IGL loci in *Pongo abelii* and *Pongo pygmaeus* reveals a compact and functionally conserved architecture, with limited copy number variation across assemblies, in contrast to the more structurally dynamic IGH locus. This reflects the well-documented evolutionary constraint imposed on light chain IG genes across mammals. Consistent with observations in human, gorilla and rhesus macaque [11, 13, 81], both loci maintain stable genomic organization with minimal evidence of large-scale structural remodeling. The appreciable heterozygosity observed across the IGK and IGL loci, despite their conserved genomic organization, indicates that diversification occurs primarily through allelic variation rather than large-scale structural change. Unlike humans, in whom the IGKV locus is divided into distinct proximal and distal clusters, orangutans show an organization more similar to that of gorillas, with a compact and continuous IGKV architecture.

### Conservation of recombination signal sequences

RSSs exhibit a high degree of sequence conservation in *Pongo* (Fig. 7). The CAC trinucleotide of the heptamer is completely conserved across all examined V-gene RSSs, consistent with its established role in efficient V(D)J recombination [82, 83]. The remaining 4 nt of the heptamer motif are also well conserved among *Pongo* V RSSs, similar to the human pattern, indicating that strong evolutionary pressure maintains this motif to ensure proper recombination. Although greater variability is observed within the nonamer, the positions 5–9 (AAACC) are well conserved in nearly all *Pongo* sequences, underscoring their critical functional role. This conservation asymmetry within the nonamer supports previous findings that highlight the importance of the AAA triplet in maintaining recombination efficiency. Overall, the *Pongo* RSS organization exhibits the same evolutionary logic observed in humans, maintaining the structural features essential for efficient V(D)J recombination [84–86].

## Conclusion

In this study, we present the first comprehensive genomic annotation of the IG loci (IGH on chromosome 15, IGK on chromosome 12, and IGL on chromosome 23) in Sumatran (*Pongo abelii*) and Bornean (*Pongo pygmaeus*) orangutans. Through the application of the IMGT-ONTOLOGY and its rigorous curation framework, we ensured accurate gene identification, functionality assessment, and full alignment with IMGT nomenclature standards. Despite being based on a limited number of individuals (two *Pongo abelii* and one *Pongo pygmaeus*), our analysis reveals a largely conserved IG loci organization with limited structural divergence between the two orangutan species. This characterization of IG loci not only enhances our understanding of immune variation in great apes but also contributes valuable additions to the IMGT reference directories, thereby establishing a strong foundation for future investigations into antibody gene evolution, diversity, and function within the Hominidae family. Moreover, these repertoires constitute a foundational framework that will be progressively expanded and refined through the annotation of future genome assemblies.

## Supplementary Material

lqag112_Supplemental_Files

## Data Availability

This study analyzed publicly available genome assemblies (GCA_028885625.3, GCA_028885655.3, GCA_028885525.2, GCA_030170355.1, GCA_030170345.1, GCA_028885685.2, and GCA_002880775.3) retrieved from the NCBI database. The newly generated IMGT-curated annotations of the IGH, IGK, and IGL loci are available through IMGT/LIGM-DB and the NCBI Third Party Annotation (TPA) database. The corresponding accession numbers for all genome assemblies and annotated sequences are provided in Table 1. The IMGT/LIGM-DB records are publicly available without restriction, and the IMGT® data are provided under the Creative Commons Attribution 4.0 International (CC BY 4.0) license.
